# Cytoplasmically localized tRNA-derived fragments inhibit translation in *Drosophila* S2 cells

**DOI:** 10.55730/1300-0152.2610

**Published:** 2022-01-17

**Authors:** Syed Muhammad HAMID, Bünyamin AKGÜL

**Affiliations:** 1 Heart Institute, Cedars-Sinai Medical Center, University of California, Los Angeles, USA; 2 Department of Molecular Biology and Genetics, İzmir Institute of Technology, İzmir, Turkey

**Keywords:** tRF, tRNA fragments, translation, Drosophila, proliferation

## Abstract

Transfer ribonucleic acids (tRNAs) serve not only as amino acid carriers during translation but also as a template for the biogenesis of short fragments that can regulate gene expression. Despite recent progress in the function of tRNA-derived fragments (tRFs), their intracellular localization, protein partners, and role in regulating translation are not well understood. We used synthetic tRFs to investigate their localization and function in *Drosophila* S2 cells. Under our experimental setting, all synthetic tRFs tested were localized at distinct sites within the cytoplasm in a similar manner in *Drosophila* S2 cells. Cytoplasmically-localized tRFs were positioned in close proximity to GW182 and XRN1 proteins. Functionally, tRFs, which slightly suppressed proliferation in S2 cells, inhibited translation without any major shift in the polysome profile. These results suggest that 5′-tRFs are cytoplasmically-localized and regulate gene expression through inhibition of translation in *Drosophila*.

## 1. Introduction

Transfer RNAs, which play a fundamental role in translation (Schimmel 2017), have recently emerged as templates for the biosynthesis of small noncoding RNAs (Gebetsberger and Polacek, 2013). The existing literature classifies tRNA-derived short RNAs into two groups: tRNA halves and tRNA-derived fragments (Keam and Hutvagner, 2015). Stress-induced tRNA halves possess a rather compact size of 30–40 nucleotides, where a mature tRNA is simply cleaved endonucleolytically into two halves. However, there appears to be a lot of heterogeneity in the size of tRFs as they can primarily stem from the 5′- or 3′-ends of mature tRNAs or 3′ of pre-tRNAs.

Small RNA-seq studies have led to the identification of a number of tRF types. 3′U tRFs contain tRNA sequences directly starting from the 3′ end of mature tRNAs and a stretch of U residues, a hallmark of an RNA polymerase III termination signal (Lee et al., 2009; Haussecker et al., 2010). Maturation of these tRFs may require Dicer (Bariarz et al., 2008), and they are localized mainly in the cytoplasm as they are quickly cleared from the nucleus (Liao et al., 2010). 3′U tRFs preferentially associate with Ago3 and Ago4 in human (Lee et al., 2009). 3′ CCA tRFs are also generated from the 3′ ends of tRNAs but do not contain any trailer sequences other than the mature tRNA sequences (Maute et al., 2013). Their processing appears to require Dicer, and they possess miRNA-like functions as they associate with the RNAi components (Haussecker et al., 2010).

The cleavage in or around the D loop of mature tRNAs generates 5′tRFs as reported by our lab and others (Cole et al., 2009; Lee et al., 2009; Kumar et al., 2014; Karaiskos et al., 2015; Olvedy et al., 2016; Göktaş et al., 2017). We reported the size range of most abundant 5′tRFs to be 26–28 nt in *Drosophila melanogaster* (Göktaş et al., 2017), but there appears to be a high heterogeneity in the average size of 5′tRFs that ranges between 19 and 26 nt (Gebetsberger and Polacek, 2013). The biogenesis of 5′tRFs is not completely understood. The 5′ processing is most likely carried out by RNase P, but there are conflicting reports on the processing of 3′ ends with respect to the involvement of Dicer in this process (Cole et al., 2009; Kumar et al., 2014; Pederson, 2010). Although a potential miRNA-like function is still in question, many labs have reported association with Ago proteins, a key component of the RNA-induced silencing complex (RISC) (Cole et al., 2009; Karaiskos et al., 2015).

There are well-documented examples of miRNAs that are derived from tRNAs [see Keam and Hutvagner, 2015 for review]. Furthermore, due to their smaller size, miRNA-like functions were attributed to various types of tRNA-derived fragments. Consequently, most studies focused on the investigation of tRF-mediated translational regulation. Existing reports show that tRFs can both activate (Kim et al., 2017) or inhibit translation (Gebetsberger et al., 2012; Sobala and Hutvagner, 2013). Although 3′-derived LeuCAG3′tsRNA activates translation through a major change in the polysome profile (Kim et al., 2017), 5′tRFs reported by Sobala and Hutvagner (2013) suppress translation of reporter constructs without a requirement for the presence of classical miRNA-binding sites. We reported recently that most 5′tRFs co-sediment with non-polysomal fractions in *Drosophila melanogaster* (Göktaş et al., 2017).

Despite great progress in the biogenesis and molecular function of 5′tRFs, there is still a lot unknown about the tRF-interacting complexes and their function. In this study, we investigated the subcellular localization and function of a *Drosophila* 5′tRF, tRF^Gly:GCC:5′:3′B^. Biotin-labelled 5′tRFs, when transfected into *Drosophila* S2 cells, were localized at specific foci in the cytoplasm rather than random diffusion. Although 5′tRFs were spatially adjacent to GW182, they do not always colocalize. Additionally, 5′ tRF transfection reduced the proliferation rate slightly affecting the global translation process as implied by polysome profiles. A 5′ tRF, when monophosphorylated at its 5′end, was capable of suppressing the translation of a reporter gene.

## 2. Materials and methods

### 2.1. S2 cell maintenance and transfection with plasmids or synthetic tRFs

S2 cells, which were generously provided by Dr. Ylva Engström of Stockholm University, were maintained in Schneider’s *Drosophila* medium at 25 ºC. pPGFP^gw^ (GW-GFP fusion), and pENTR^pcm^-pAWR (PCM-RFP fusion) plasmids were kindly provided by Prof. Dr. Andwer Simmonds of University of Alberta (Scheneider et al., 2006). Synthetic tRFs were transfected into S2 cells at a concentration of 100–500 nmol using the Metafectene pro (Biontex) transfection kit according to the manufacturer’s instructions. The sequences of tRFs are presented in Table.

### 2.2. Fluorescence microscopy

S2 cells were seeded on glass cover slips. Twenty-four hours after transfection (unless specified), cells were fixed with 4% paraformaldehyde for 1 h at room temperature. Cells were permeabilized with 0.1% triton X-100 in PBS for five minutes, rinsed with PBS, and blocked with 1% BSA in PBS for five minutes. Cells were incubated with primary antibody streptavidin or anti-digoxigenin for 1 h at room temperature, washed with PBS three times, and incubated with secondary antibody (AlexaFlor 488 or 594) for 1 h at room temperature. Cells were mounted using Flourshield mounting medium with DAPI. Images were taken with a Leica DMIL florescent microscope. To determine the localization and the number of tRF foci, 200–300 cells (268 cells on average) were screened under the microscope for the localization and the number of tRFs per cell.

### 2.3. Cell proliferation assay

S2 cells were seeded on 12-well plates at a density of 1 million cells per well 24 h prior to transfection. Twenty-four hours after transfection, cells were scraped and seeded on 96 well plates at a density of ten thousand cells per well. Cell proliferation was measured using XTT cell proliferation assay kit (Biological Industries) according to the manufacturer’s protocol after 24, 48 and 72 h of transfection.

### 2.4. Polysome profiling

Polysome profiles were obtained according to a previously published procedure (Göktaş et al., 2017). Briefly, cell lysis (3×10^7^ cells) was carried out in 5 mL lysis buffer [(100 mM NaCl, 10 mM MgCl_2_, 30 mM Tris-HCl (pH 7), 1% Triton X-100, 1% NaDOC, 100 μg/mL cycloheximide (Applichem) and 30 U/mL SUPERase.In RNase Inhibitor (Ambion)], and the lysate was incubated on ice for 8 min. The cell debris and nuclei were removed by centrifuging the homogenates at 12,000 *g* at 4 °C for 8 min. Two-mL supernatant was loaded onto 5%–70% (w/v) sucrose gradients [100 mM NaCl, 10 mM MgCl_2_, 30 mM Tris-HCl (pH 7), 200 U SUPERase.IN RNase inhibitor (Ambion)] and centrifuged at 27,000 rpm for 2 h 55 min at 4 °C in a Beckman SW28 rotor. Fractions were collected using Teledyne ISCO’s density gradient fractionation system (NE, USA) while recording the absorbance at A_254_ to obtain the polysome profiles.

### 2.5. Dual luciferase assay

S2 cells were seeded on twelve-well plates at a density of one million cells per well. Next morning, cells were transfected with 2 μg of pAct-Luciferase vector alone or along with 300 pmol of indicated tRFs using Metafectene Pro (Biontex) according to manufacturer’s instructions. Twenty-four hours after transfection, cells were lysed using Promega passive lysis buffer, and luciferase activity was measured on VarioScan (Thermo) using Promega luciferase assay kit according to manufacturer’s recommendations.

### 2.6. Statistical analyses

All experiments were carried out in triplicates unless indicated. Values are indicated in mean and standard deviation. Student t test was used to assess the statistical significance of two data point where p ≤ 0.01 was considered statistically significant.

## 3. Results

**Figure 1:**
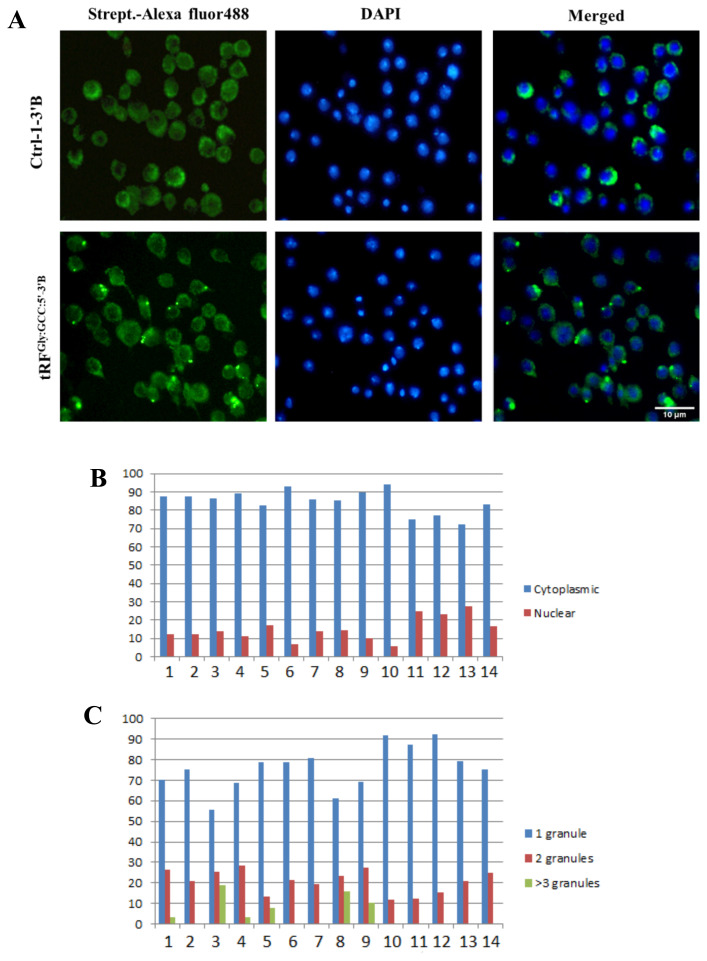
tRF^Gly:GCC:5′:3′B^ localizes to cytoplasmic granules. A. Fluorescence microscopy of control- and tRF-transfected cells. B. Distribution of tRFs in the nucleus and cytoplasm. Twenty-four hour post-transfection, the localization, and the number of AlexaFluor488 positive foci were determined by counting at least 200 cells (200–350 cells, 268 cells on average) from three different biological replicates. The cytoplasmic and nuclear percentage is presented in a graph. C. The percentage of the number of tRF foci per cell. The number of tRF foci per cell was calculated as in Panel B. The percentage of cells with 1, 2, or >3 granules was presented in a graph. 1. tRF^Ala:AGC:5′:P:3′B^ 2. tRF^Pro:AGG:5′:P:3′B^ 3. tRF^Cys:GCA:5′:P:3′B^ 4. tRF^Gly:GCC:5′:TOG:3′B^ 5. tRF1001 6. tRF^Gly:GCC:3′:P:3′B^ 7. tRF^Gly:GCC:3′:3′B^ 8. tRF^Gly:GCC:5′:P:3′B^ 9. tRF^Gly:GCC:5′:P:Int.B^ 10. tRF^Gly:GCC:5′:3′B^ 11. Ctrl:3:P:Intr.B 12. Ctrl:3:3′B 13. Ctrl:2:5′P:3′B 14. Ctrl:1:3′B.

**Figure 2:**
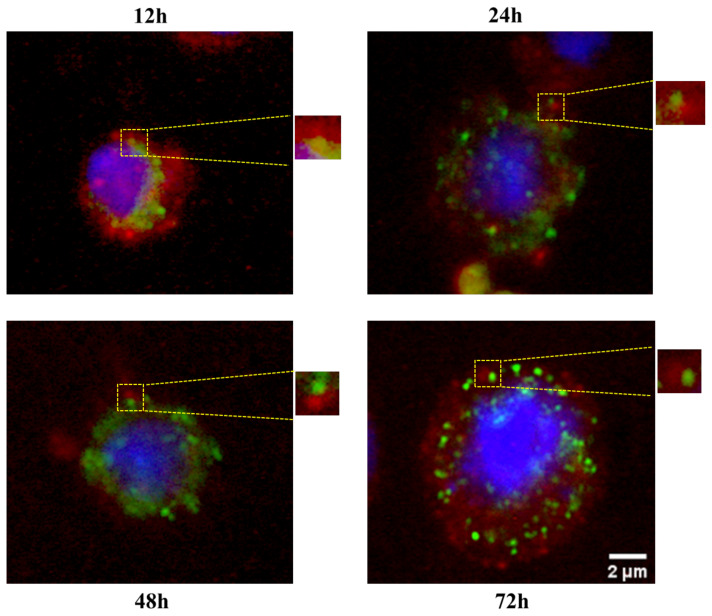
Colocalization of tRF^Gly:GCC:5′:3′B^ with GFP-GW182 fusion protein. S2 cells were cotransfected with tRF^Gly:GCC:5′:3′B^ and the plasmid pPGFP^gw^ that contains a P body marker GFP-GW182. Fluorescein images were acquired 12–72h posttransfection. The nucleus was stained with DAPI (blue color). Shown is a representative of three replicates.

**Figure 3:**
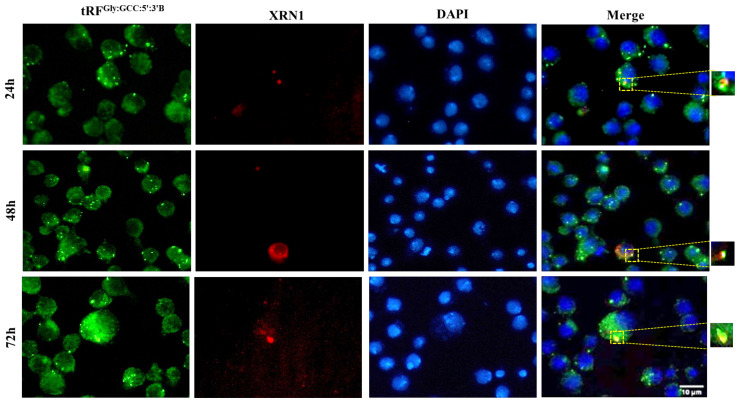
Colocalization of tRF^Gly:GCC:5′:3′B^ with XRN1. The experiment was carried out essentially as explained in Figure 2 but cells were co-transfected with XRN1. Shown is a representative of three replicates.

tRF^Gly:GCC:5^ localizes adjacent to GW182 and XRN1 in the cytoplasm: We have previously reported that tRFs are differentially expressed during early development in *Drosophila* (Göktaş et al., 2018). tRF^Gly:GCC:5^, which is the most abundant tRF in *Drosophila*, is expressed in 1–24h embryos, adults and S2 cells. However, the function and localization of tRF^Gly:GCC:5^ at the cellular level is unknown. tRFs can localize to various subcellular sites, such as nucleus (Kumar et al., 2014), cytoplasm (Lee et al., 2009; Haussecker et al., 2010; Liao et al., 2010), or exosomes (Vojteck et al., 2014). Since the subcellular localization can provide insight into potential interacting complexes and/or function, we first checked the subcellular localization of tRF^Gly:GCC:5′:3′B^. Biotinylated-tRF^Gly:GCC:5′:3′B^ primarily localized to the cytoplasm (Figure 1A). Additionally, we investigated the localization of a number of different tRFs, both 5′-monophosphorylated and nonphosphorylated forms, to check whether this localization or function requires a 5′-monophosphate as reported for 5′-tiRNAs (Emara et al., 2010). Our data indicate an overwhelming localization in the cytoplasm of all tRFs tested irrespective of their 5′-phosphorylation status (Figure 1B). We then counted the number of granules per cell to examine whether there are any differences in the number of granules formed by different tRFs. Typically, we observed a singular or dual (relatively less) granule in each cell irrespective of the identity of tRF (Figure 1C). Rather than diffusing through the cytoplasm, we noted localization at discreet sites. 5′-monopohosphorylated tRFs produced relatively weaker signals. Thus, we used nonphosphorylated forms for the localization studies as we reported their stable presence posttransfection in S2 cells (Göktaş et al., 2017). There are two well-known cytoplasmic structures associated with RNA metabolism: P bodies and stress granules (Balagopal and Parker, 2009). As potential miRNA-like functions (Haussecker et al., 2010; Maute et al., 2013) and stress granule formation (Emara et al., 2010) were attributed to tRFs and tiRNAs, respectively, we examined whether tRF^Gly:GCC:5′:3′B^ colocalizes with these structures in the cytoplasm. To this extent, we first co-transfected S2 cells with GFP-tagged GW182, a component of P bodies (Balagopal and Parker, 2009), and biotinylated tRF^Gly:GCC:5′:3′B^. Interestingly, we observed a very low level of transfection efficiency with the GW182 plasmid. Although tRF did not precisely colocalize with GW182, they were adjacent to each other (Figure 2). We, then, examined the colocalization pattern of stress granules and tRFs by cotransfecting S2 cells with biotinylated tRF^Gly:GCC:5′:3′B^ and RFP-tagged-XRN1 (Pacman), a component of stress granules. Our data suggest that their localization sites overlap (Figure 3).

**Figure 4:**
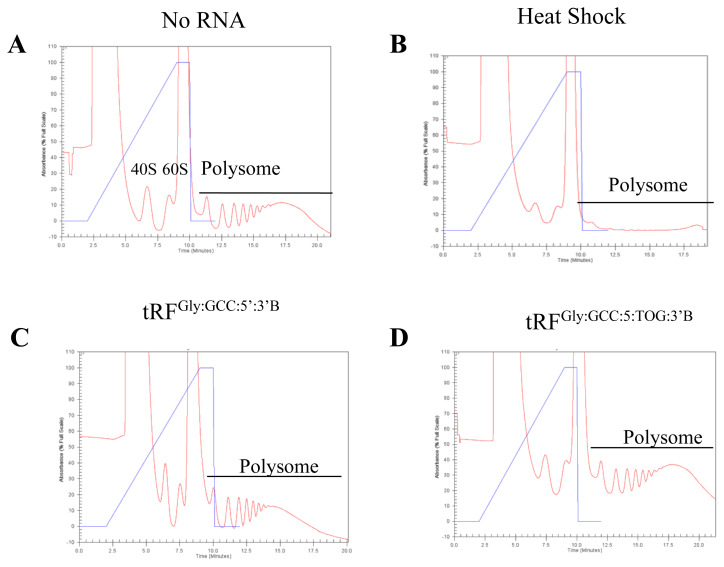
5′-tRFs slightly affect the polysome assembly in S2 cells. S2 cells were transfected with 500 nm of indicated synthetic tRFs. Cytoplasmic cell lysates were prepared after over-night transfection of three biological replicates. Equal volumes of O.D 260 were mixed from replicated before being loaded on 5%–70% sucrose gradients and centrifuged at 26,000 g for 3 h. Polysome profiles were observed using a density gradient fractionating system (ISCO).

We examined the polysome profiles of S2 cells transfected with tRFs to investigate whether tRFs target polysomes and causes a global translational regulation in *Drosophila*. To this extent, we first obtained the polysome profile of untransfected control S2 cells, which displayed a proportional ratio of 40S, 60S, monosomes, and polysome (Figure 4A, label 40S, 60S on the figure). A high volume of polysomal fraction was a sign of efficient translation. We also examined the polysome profile of heat-shock-treated S2 cells to show that global translational suppression, e.g., by heat shock, causes a major reduction in the polysome volume (Figure 4B). We then checked the ability of two different versions of the synthetic tRF^Gly:GCC:5^ to cause global translational block. The biotinylated tRF^Gly:GCC:5′:3′B^ caused a slight reduction in the volume of polysomes (Figure 4C). Since terminal oligoguanine (TOG) motifs (4–5 guanine nucleotides) are required for translational block by angiogenin-induced tRNA fragments (Ivanov et al., 2011), we also examined whether a synthetic tRF^Gly:GCC:5^ with a TOG affects the polysome status. Thus, we used a synthetic tRF^Gly:GCC:5′:TOG:3′B^ that carries 4 guanine residues at its 5′ terminal. Interestingly, we detected a slight increase in the polysome volume when S2 cells were transfected with this tRF (Figure 4D).

tRF^Gly:GCC:5′:3′B^ causes a slight shift in the polysome profile: Although not applicable to all tRFs, there are examples in which a specific tRF can regulate translation by interfering with constituents of polysomes (Gebestberger et al., 2012; Kim et al. 2017). In fact, the targeting by a tRF of an rRNA has been reported to cause a major shift in the polysome profile in the hepatocellular carcinoma model in mice (Sobala and Hutvagner, 2013).

**Figure 5:**
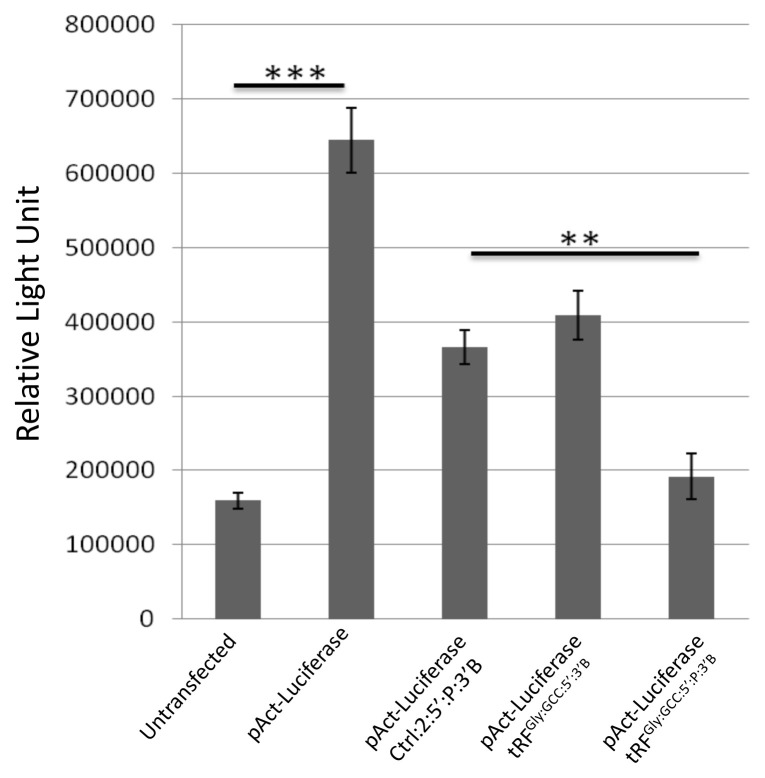
5′-monophosphorylated tRF^Gly:GCC:5′:P:3′B^ causes translational repression of a reporter construct. pAct-Luciferase was cotransfected, in triplicates, into S2 cell with Ctrl:2:5′:P:3′B, tRF^Gly:GCC:5′:3′B^ or tRF^Gly:GCC:5′:P:3′B^, and luciferase activity was measured using dual luciferase assay (Promega) 48 h posttransfection. Untransfected control cells. *** p < 0.005 (untransfected versus pAct-luciferase); ** p < 0.05 (Ctrl:2:5′:P:3′B versus tRF^Gly:GCC:5′:P:3′B^ )

Although a slight shift in the polysome profile could be an indicative of a perturbation in global translation, we wanted to collect supportive data by examining the translation efficiency of individual mRNAs. Previously, some tRFs were shown to regulate translation without a requirement for a binding site on a potential target mRNA in human cells (Sobala and Hutvagner, 2013), possibly through modulation of translation elongation. Thus, we examined whether or not tRF^Gly:GCC:5^ can regulate the translation of a luciferase reporter gene in a similar manner in *Drosophila*. To this extent, we first transfected a luciferase construct into S2 cells, which caused an increased luciferase activity as detected by a dual luciferase reporter assay (Figure 5). 5′-nonphosphorylated tRF^Gly:GCC:5′:3′B^ reduced the luciferase activity to an extent similar to that of a control short RNA. However, 5′-monophosphorylated tRF^Gly:GCC:5′:P:3′B^ further reduced the luciferase activity by nearly 50% compared to the control tRF (p < 0.05, Ctrl:2:5′:P:3′B vs tRF^Gly:GCC:5′:P-3′B^).

**Figure 6:**
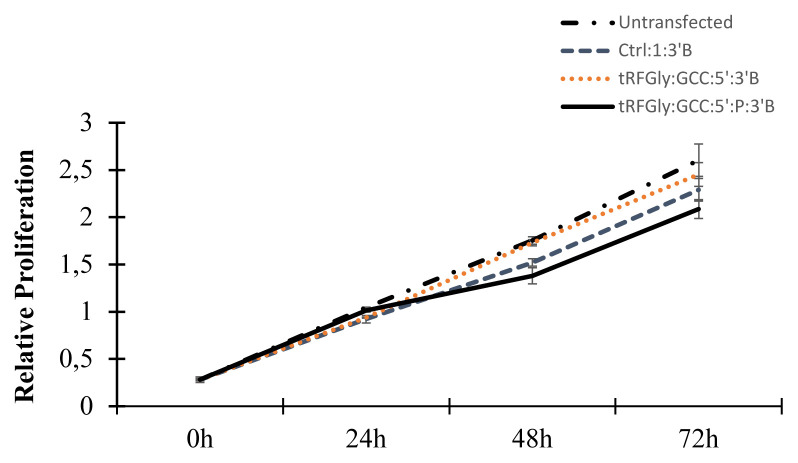
tRF^Gly:GCC:5′:P:3′B^ reduces cell proliferation in S2 cells. S2 cells were seeded on 12-well plates at a density of 1 million cells per well 24 h prior to transfection. Twenty-four hours after transfection, cells were scraped and seeded on a 96 well plate at a density of ten thousand cells per well. An XTT cell proliferation assay kit (Biological Industries) was used according to manufacturer’s protocol to measure the proliferation rate. The proliferation rate was plotted relative to the untransfected cells. The experiment was done in triplicates and repeated twice. Error bars show SEM. P value was calculated at the 72 h data point. P < 0.02 (Ctrl:1:3′B versus tRF^Gly:GCC:5′:P:3′B^).

tRF^Gly:GCC:5′:P:3′B^ slows down proliferation in S2 cells: Previous reports suggest that certain tRFs regulate apoptosis and proliferation in a variety of eukaryotic cell types (Haussecker et al., 2010; Maute et al., 2013; Olvedy et al., 2016). Thus, we checked the effect of tRF^Gly:GCC:5^ on the proliferation rate of S2 cells. Untransfected cells had the highest rate of proliferation as expected (Figure 6). However the cells transfected with tRF^Gly:GCC:5′:3′B^ without a phosphate group at its 5′end had a better proliferation rate compared to the control scrambled RNA. Previous studies on 5′-tiRNAs (tRNA-derived stress induced RNAs) showed that synthetic 5′-tiRNAs are incapable of inducing stress granule formation in the absence of a 5′-monophosphate group (Emara et al., 2010). Thus, we also examined the proliferative state of S2 cells when transfected with 5′-mono-phosphorylated tRF^Gly:GCC:5′:P:3′B^. Our data showed that 5′-monophosphorylated tRF^Gly:GCC:5′:P:3′B^ causes a decrease in the proliferation rate (p < 0.025, at 72h, Ctrl-1–3′B versus tRF^Gly:GCC:5′:P:3′B^). Transfection of tRF^Gly:GCC:5′:3′B^ or its monophosphorylated form up to forty-eight hours did not lead to any detectable cell death in S2 cells (data not shown).

## 4. Discussion

In the present work, we provide interesting data with respect to the intracellular localization of tRFs and their potential function in gene regulation. Our data show that transfected synthetic tRFs localize to specific sites in the cytoplasm in close proximity with GW182 and within overlapping sites of XRN1. Additionally, we provide evidence for tRF-mediated translational regulation of a reporter construct.

Although the cytoplasmic localization and interacting proteins of tRNA halves are relatively well-characterized (Thompson and Parker, 2009), the biogenesis, localization, and molecular function of tRFs are still under investigation. The existing evidence points to the cytoplasmic localization of tRFs in mammals (Haussecker et al., 2010; Liao et al., 2010). Interestingly, although 3′tRFs are localized in the cytoplasm, the majority of 5′tRFs has been reported to localize in the nucleus in HeLa cells (Kumar et al., 2014; Kumar et al., 2015). In *Tetrahymena*, 3′tRFs are bound to Ago/Piwi protein Twi12 in the nucleus (Couvillion et al., 2012). We reported previously nonpolysomal association of 5′tRFs (Göktaş et al. 2017; Cosacak et al. 2018), suggesting the cytoplasmic localization of at least a fraction of them in *Drosophila melanogaster* embryos and S2 cells. In this study, we used synthetic and 3′-biotinylated tRFs over-expressed in S2 cells to quantitatively measure the intracellular location of tRFs as 3′-biotinylation does not appear to interfere with the biological function of tRFs (Goodarzi et al., 2015). Our data suggest that 5′tRFs are overwhelmingly localized to the cytoplasm under our experimental setting (Figure 1). The use of synthetic tRFs has several advantages to examine the cellular location of tRFs. Firstly, it facilitates the convenient distinction from mature tRNAs, which would generate false-positive signals in a hybridization-based approach. Secondly, it makes it possible to amplify the intensity of the signal especially when the copy number of the tRF is low. Thirdly and more importantly, mutational analysis can be carried out with synthetic tRFs to probe into mechanistic details. One major disadvantage of synthetic tRFs, on the other hand, is that supraphysiological conditions require more careful interpretation of the data, mostly requiring validation with endogenous tRFs. Unfortunately, the potential cross hybridization with the mature tRNA sequences presents itself as a major challenge to study endogenous tRFs through hybridization-based intracellular localization studies.

The cytoplasmic localization of synthetic tRFs in distinct foci has prompted us to further characterize the putative complexes that might house tRFs. Cytoplasmic localization suggests that tRFs should regulate gene expression post-transcriptionally, probably at the levels of RNA metabolism or translation rather than transcriptional or epigenetic regulation. There are three cytoplasmic complexes associated with posttranscriptional gene regulation, polysomes, P bodies, or stress granules (Balagopal and Parker, 2009). Due to the small size of tRFs, miRNA-like functions have been attributed to tRFs. Thus, a number of studies have focused on potential interaction between tRFs, AGO proteins and polysomes. The existing evidence suggests that tRFs associate with AGO proteins in ciliate protozoa (Couvillion et al., 2012), silkworm (Nie et al., 2013), plants (Loss-Morais et al., 2013), *Drosophila* (Karaiskos et al., 2015), mouse (Li et al., 2012) and human (Cole et al., 2009; Haussecker et al., 2010; Telonis et al., 2015). Accordingly, two studies have reported polysome association of tRFs (Gebestberger et al., 2012; Göktaş et al., 2017). However, our deep-sequencing data from unfractionated and fractionated 0–1 and 7–8h *Drosophila* embryos showed that 5′tRFs overwhelmingly exist in the mRNP fraction, which contains mRNP complexes and free RNAs (Göktaş et al., 2017). Thus, we turned our attention to P bodies and stress granules as alternative cytoplasmic locations. GW182, which is mainly involved in miRNA function, is predominantly found in P bodies whereas XRN1 is a component of both P bodies and stress granules (Eystathioy et al., 2003; Sheth and Parker, 2003). Our data suggest that synthetic tRFs localize to specific sites in close proximity with GW182 that includes XRN1 as well (Figure 2 and 3). XRN1 is known to be a component of both P bodies and stress granules (Sheth and Parker, 2003). No difference in the number of XRN1-tRF containing granules under stress conditions (e.g., heat shock) (data not shown) supports the notion that the transfected synthetic tRFs are more likely to be part of P bodies rather than stress granules. There are at least two reasons as to why tRFs could potentially localize to sites in close proximity to P bodies: (1) tRF-containing regulatory complexes could be carrying target RNAs into P bodies (or nearby complexes) as part of gene regulation, (2) alternatively tRFs themselves could be transported to P bodies as part of their metabolism. Although more experiments are required to conclusively demonstrate the association of tRFs with P bodies, the data collected under our experimental setting points to a potential association between tRFs and P bodies.

Although the majority of tRFs sediment with the nonpolysomal fraction in *Drosophila melanogaster* (Göktaş et al., 2017), tRFs could still potentially regulate translation at the preinitiation or initiation state. Angiogenin-induced tRNA halves were shown to inhibit translation (Ivanov et al., 2011). In a similar manner, tRFs were also shown to inhibit translation in a cap-dependent manner in human cells (Sobala and Hutvagner, 2013). Interestingly, a 3′ tRF (LeuCAG3′tsRNA) was reported to enhance translation by directly binding to ribosomal protein mRNAs in a patient-derived orthotopic hepatocellular carcinoma model in mice (Kim et al., 2017). Accordingly, LeuCAG3′tsRNA inhibition results in the disruption of ribosome biogenesis and a major shift in the polysome profile. Such an extensive change in the polysome distribution would be expected to have an influence on global translation regulation as well. To this extent, we first checked whether the transfection of synthetic tRFs modulates global translation in *Drosophila melanogaster*. We detected a slight decrease in the polysome volume under the experimental setting (Figure 4). A similar observation was reported in *Drosophila* S2 cells (Luo et al., 2018). We also looked at the ability of tRFs to regulate the translation of an individual reporter mRNA. Interestingly, transfection of a synthetic tRF decreased reporter gene activity in S2 cells (Figure 5). Since the reporter construct did not contain any sequence that could serve as a binding site for the transfected tRF, it appears that tRF-mediated translation inhibition might not require extensive complementarity between the tRF and its target, at least for the synthetic tRF tested. This observation is in consistency with the translational repression modulated by tRNA-derived stress-induced fragments, which inhibit protein synthesis, without requiring a complementary target site, by displacing eIF4G/eIF4A from mRNAs (Ivanov et al., 2011). Existing evidence suggests that there is a lot of heterogeneity in the sequence of tRFs and their interaction with eIF4 (Xie et al., 2020). Thus, further experiments are required to elucidate if tRF-mediated translational regulation involves eIF4 in *Drosophila*. We cannot conclusively state if there is any relationship between the 5′-monophosphorylation state and functionality. However, the translational block by the 5′-monophosphorylated tRF is in agreement with a study reported by Sobala and Hutvagner (2013). However, a recent report by Luo et al. (2018) suggests that tRFs preferentially suppress translation through antisense pairing, providing an alternative hypothesis for tRF-mediated translational regulation. Thus, more studies are required to uncover the molecular mechanisms that underlie the macromolecular interactions and cellular functions of tRFs.

## Figures and Tables

**Table:** Nucleotide sequences of synthetic tRNA-derived fragments. The internal biotin site is shown with “1”. tRF^Aaa:BBB:C′:D′B^ nomenclature is used to represent different types of biosynthetic tRFs where Aaa refers to the type of tRNA (e.g., Glycine), BBB to the isotype of tRNA (e.g., GCC-codon carrying), C′ to the origin of tRF (e.g., 5′- or 3′-derived), and D′B to the location of the biotin residue (e.g., 3′-derived or internal-Int). P is used to refer to 5′ mono-phosphorylated tRFs.

No.	Name	Flybase ID	Sequence 5′-3′
**1**	tRF1001		**GAA GCG GGU GCU CUU AUU U**
**2**	tRF^Gly:GCC:5′:3: ′B^	CR31667	**GCA UCG GUG GUU CAG UGG UAG AAU GC**
**3**	tRF^Gly:GCC:5′:P:Int.B^	CR31667	**GCA UCG GUG GUU CAG UGG UAG AAU 1GC**
**4**	tRF^Gly:GCC:5′:P:3′B^	CR31667	**GCA UCG GUG GUU CAG UGG UAG AAU GC**
**5**	tRF^Gly:GCC:5′:TOG:3′B^	CR31667	**GGG GGU GUG GUU CAG UGG UAG AAU GC**
**6**	tRF^Gly:GCC:3′:3′B^	CR31667	**GGG UUC GAU UCC CGG CCG AUG CAC CA**
**7**	tRF^Gly:GCC:3′:P:3′B^	CR31667	**GGG UUC GAU UCC CGG CCG AUG CAC CA**
**8**	Ctrl:1:3′B		**GCA UCG GCG UAG CCA CCA AGU UAG AA**
**9**	Ctrl:2:5′:P:3′B		**GUU CGA UCG UAG AGU CCA AGU UAC AU**
**10**	Ctrl:3:3′B		**GCA UUC ACU UGG AUA GUA AAU CCA AG**
**11**	Ctrl:3:P:Intr.B		**GCA UUC ACU UGG AUA GUA AAU CCA 1AG**
**12**	tRF^Ala:AGC:5′:P:3′B^	CR31577	**GGG GAU GUA GCU CAG AUG GUA GAG C**
**13**	tRF^Cys:GCA:5′:P:3′B^	CR32289	**GGG GAU AUA GCU CAG UGG UAG AGC AUU C**
**14**	tRF^Pro:AGG:5′:P:3′B^	CR31979	**GGC UCG UUG GUC UAG GGG UAU GAU UUC**
